# Health-related quality of life in patients undergoing adrenalectomy: report from a Swedish National Audit

**DOI:** 10.1007/s00423-019-01844-4

**Published:** 2019-11-26

**Authors:** Lo Hallin Thompson, Erik Nordenström, Martin Almquist, Anders Bergenfelz

**Affiliations:** Department of Surgery, Skåne University Hospital, 22185 Lund, Sweden

**Keywords:** Adrenalectomy, Quality of life, SF-36, Adrenal hyperfunction, Adrenal tumour

## Abstract

**Purpose:**

The aim of the study was to examine subjective health-related quality of life (HRQoL) in patients undergoing adrenalectomy.

**Methods:**

The study included patients scheduled for adrenalectomy 2014–2017 after giving informed consent. The SF-36 questionnaire was administrated before operation and 1 year postoperatively. Results were compared with published normative values in Sweden.

**Results:**

Some 50 patients were included. SF-36 scores for the whole cohort improved significantly after adrenalectomy in all dimensions except for bodily pain. Compared with the general Swedish population, the patients reported a significantly reduced HRQoL before and after adrenalectomy in all domains except for bodily pain postoperatively. Patients with benign functional tumours had lower HRQoL in physical domains before adrenalectomy than patients with benign non-functional tumours; Physical Component Summary (PCS), median 33.1 (range 17.1–62.9) vs. 44.2 (20.0–66.5), *p* = 0.018. Postoperatively, HRQoL was similar in the two groups of patients. Patients with benign functional tumours reported significantly improved HRQoL in all dimensions after adrenalectomy: PCS 33.1 (17.1–62.9) preoperatively vs. 47.6 (19.8-57.3) postoperatively, *p* = 0.005; Mental Component Summary (MCS) 33.8 (11.8–62.0) preoperatively vs. 52.7 (16.4–59.8) postoperatively, *p* = 0.004. These improvements were not seen in patients with benign non-functional or malignant tumours. Patients with malignant tumours reported no difference in SF-36 scores before or after adrenalectomy compared with patients with benign non-functional tumours.

**Conclusions:**

Adrenalectomy improved HRQoL in patients with benign functional tumours. Adrenalectomy did not improve HRQoL in patients with benign non-functional tumours or in patients with malignant tumours.

**Electronic supplementary material:**

The online version of this article (10.1007/s00423-019-01844-4) contains supplementary material, which is available to authorized users.

## Introduction

An increasing number of patients are being diagnosed with adrenal incidentaloma due to more frequent use and improvement in imaging. Adrenal incidentalomas increase with age [1], and the prevalence of incidentaloma in routine imaging has been reported to be 1–4% [2, 3]. Annual rates of adrenalectomy have nearly doubled since 1998 in many countries [4]. This increase in adrenal surgery has not been followed by an increase in detection of malignant adrenal disease [5], which could indicate that considerable number of patients undergo unnecessary surgery. Adrenal cortical adenoma is the most frequent pathological finding in incidentalomas [6]. Most of these benign tumours are non-functional cortical adenomas [7], but it has been suggested that these tumours can be associated with poor health outcomes [8–10], and possibly ameliorated by surgery[11].

Patients with hormonally inactive, small tumours with low density on computed tomography (CT) are usually not treated with surgery. Treatment for growing hormonally inactive tumours with low density on CT or small tumours with high density on CT is still debated [12].

A, sometimes, neglected aspect in patients with adrenal tumours is health-related quality of life (HRQoL) [13]. Quality of life (QoL) evaluation describes the subjective perception of the patient [14]. HRQoL is to which degree the illness or treatment affects the life of the patient [15]. There is a growing interest in QoL evaluation in patients with adrenal disease but data are very scant [13]. Previous studies have indicated reduced HRQoL in patients with adrenal tumours independent of diagnosis [16]. Kastelan et al. demonstrated impaired QoL in patients with non-functional adrenal incidentalomas compared with controls [17]. Previous reports that focused on patients with functional tumours reported an improvement in HRQoL after adrenalectomy [18].

In the view of these data, the effect of adrenalectomy on HRQoL in different adrenal conditions is of considerable interest. The aim of this study was to evaluate HRQoL in all patients undergoing adrenalectomy and to investigate the difference in the effect of adrenalectomy for patients with functioning, non-functioning and malignant tumours.

## Material and methods

### Study design and population

Patients undergoing unilateral adrenalectomy, at the Department of Surgery, Skåne University Hospital-Lund November 2013–March 2017 were included in the study after information and written consent. This was as part of a quality control program within the Scandinavian Quality Register for Thyroid, Parathyroid and Adrenal Surgery (SQRTPA) and thus ethical clearance was waived [19]. Patients were invited to participate in the study at their preoperative visit. They were included after returning two complete questionnaires administrated by mail 2 weeks before and at 1 year after surgery. Up to one remainder was sent to non-responders. Results were compared with values from a large sample of the general Swedish population [20].

### Variables

Data extraction from SQRTPA included age, sex, indication for surgery (clinical syndrome of hormonal excess, suspected malignancy on the basis of radiological examination, preoperative diagnosed metastases, tumour size) and histopathology (adrenal cortical adenoma, adrenal cortical hyperplasia, phaeochromocytoma, myelolipoma, metastasis and other benign tumours). The transabdominal, laparoscopic robot-assisted technique was used in all patients.

### Health-related quality of Life

SF-36 (36-Item Short Form Health Survey) was used to evaluate patients’ subjective perception of HRQoL. SF-36 is a self-administrated validated generic questionnaire that has been used extensively previously [21–26]. It consists of 36 questions evaluating both physical and mental health in eight health domains; physical functioning (PF), bodily pain (BP), role limitations due to physical health problems (role-physical, RP), role limitations due to personal or emotional problems (role-emotional, RE), emotional wellbeing (mental health, MH), social functioning (SF), energy or fatigue (vitality, VT) and general health perception (general health, GH). Scores range from 0 to 100 with higher values representing better QoL. The scores may be aggregated into two summary measures; Physical Component Summary (PCS) and Mental Component Summary (MCS) [27].

The primary outcomes were differences in HRQoL before and after adrenalectomy assessed by SF-36.

HRQoL depending on indication for surgery, hormonal hypersecretion and histopathology was also reported.

### Statistics

Descriptive statistics are presented as number with valid percentage and mean and standard deviation (SD) or median and range, as appropriate. Preoperative and postoperative SF-36 scores were assessed using paired samples *t* test or Wilcoxon test where appropriate. Differences between different patient groups were evaluated using independent samples *t* test or Mann-Whitney *U* test.

Missing values were handled according to the SF-36 manual and substituted as a person-specific estimate when at least 50% of the items in a specific domain were answered [20].

Minimally important difference (MID), defined as the smallest difference in score which patients perceived as beneficial or harmful, was used to determine clinical significance [28]. According to Osoba et al., who used the questionnaire QLQ-C30 in patients with chemotherapy for breast cancer and small-cell lung cancer, differences of 5–10 points between groups on a 100-point scale were interpreted as clinically small difference, 10–20 points as moderate difference and > 20 points as large difference [29].

Analyses were conducted using IBM SPSS Statistics 25 for Windows (IBM Corporation, Armonk, NY, USA) and STATA/SE 13.1 for Mac (StataCorpLp, College Station, USA). A *p* value < 0.05 was considered significant.

## Results

Some 161 patients underwent adrenalectomy during this time period. Fifty-nine patients returned questionnaires before adrenalectomy and at follow-up 1 year after operation. Nine patients were excluded; the first questionnaire was dated after adrenalectomy (six patients), two patients had a poor health status and were not able to complete the 1-year follow-up form, and one patient had a second operation on the contralateral adrenal before the one-year follow up.

**Fig 1:**
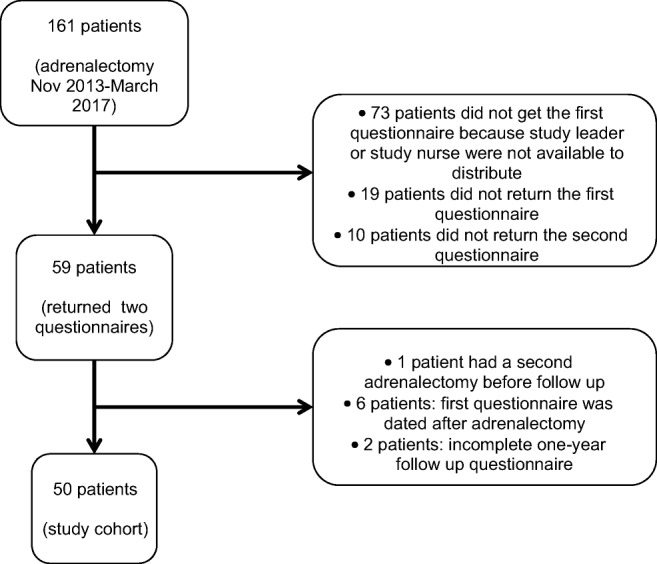
Flowchart of cohort creation

The study flow chart is shown in Fig. 1. In all, 50 patients were included in the study. Preoperative characteristics of the cohort are summarized in Table 1. No differences were detected between the cohort and non-included patients (Supplementary Table 1).

**Table 1:** Patient and tumour characteristics. Operative outcome

		*n* = 50	(%)
Age (median (range), year)	64 (20–84)		
Female		26	(52)
Indication for surgery			
Clinical syndrome of hormonal excess		27	(54)
Cortisol		12	(24)
Aldosterone		5	(10)
Catecholamines		10	(20)
Suspected malignancy on radiology		4	(8)
Metastasis		7	(14)
Tumour size only (≥ 40 mm)		12	(24)
Histopathology			
Adrenal cortical adenoma		26	(52)
Adrenal cortical hyperplasia		2	(4)
Phaeochromocytoma		12	(24)
Myelolipoma		2	(4)
Metastasis		6	(12)
Other benign tumour		2	(4)
Operative outcome			
Length of hospital stay (mean, days)	2.7		
Reoperation		0	(0)
Complication		2	(4)

The median age was 64 (range 20–84) years, and 26 (52%) patients were women. Indication for operation could be one or several. The most common indication was a clinical syndrome of hormonal excess; 12 (24%) patients had hormonal excess of cortisol, 10 (20%) patients had hypersecretion of cathecholamines, and 5 (10%) patients had primary aldosteronism. In 4 (8%) patients, malignancy was suspected on the basis of radiological examination (CT or MRI). Seven (14%) patients had preoperatively diagnosed metastasis and 12 (24%) patients underwent surgery because of a tumour size larger than 40 mm as the sole surgical indication. The most frequent histopathological diagnoses were adrenal cortical adenoma, 26 (52%) patients, and phaeochromocytoma, 12 (24%) patients (Table 1). There were six patients with malignant tumour (metastasis from renal cancer four patients, metastasis from rectal cancer one patient and metastasis from sarcoma one patient). Mean length of hospital stay was 2.7 days, there was no reoperation and 2 (4%) patients had at least one complication.

### Analysis of the whole cohort

SF-36 scores for the whole cohort before and at 1 year after adrenalectomy and compared with published normative values in Sweden [20] are shown in Table 2. The statistical precision of our findings was quantified (Supplementary Figure 1). Patients reported improved HRQoL after adrenalectomy in all dimensions except for bodily pain, mean ± SD 63.1 ± 30.7 preoperatively vs. 69.0 ± 31.5 postoperatively, *p* = 0.15. Compared with the general Swedish population, the patients reported significantly lower SF-36 scores both before and after adrenalectomy in all domains except for bodily pain postoperatively, which was no different compared with the general Swedish population (Table 2).

**Table 2:** SF-36 scores before and at 1 year after adrenalectomy (*p* value^c^) and compared with the general Swedish population (*p* value^b^). Mean and standard deviations are shown

	General population* *n* = 8930	Preoperative *n* = 50	*p* value^b^	Postoperative *n* = 50	*p* value^b^	*p* value^c^
Physical functioning	87.9 ± 19.6	64.0 ± 28.9	< 0.001^a^	72.4 ± 28.8	< 0.001^a^	0.004^a^
Role-physical	83.2 ± 31.8	46.9 ± 46.1	< 0.001^a^	65.3 ± 43.5	< 0.001^a^	0.002^a^
Bodily pain	74.8 ± 26.1	63.1 ± 30.7	0.002^a^	69.0 ± 31.5	0.118	0.150
General health	75.8 ± 22.2	56.0 ± 26.0	< 0.001^a^	62.2 ± 26.5	< 0.001^a^	0.015^a^
Vitality	68.8 ± 22.8	46.6 ± 30.2	< 0.001^a^	57.9 ± 27.7	< 0.001^a^	0.002^a^
Social functioning	88.6 ± 20.3	66.3 ± 31.2	< 0.001^a^	78.0 ± 32.4	< 0.001^a^	0.017^a^
Role-emotional	85.7 ± 29.2	49.3 ± 45.6	< 0.001^a^	66.0 ± 45.3	< 0.001^a^	0.015^a^
Mental health	80.9 ± 18.9	63.0 ± 28.2	< 0.001^a^	74.9 ± 23.0	0.025^a^	< 0.001^a^
Physical Component Summary		41.2 ± 13.2		44.0 ± 13.2		0.050
Mental Component Summary		39.3 ± 16.1		45.8 ± 13.9		0.001^a^

*Normative values from the general Swedish population published in the Swedish manual and interpretation guide of SF 36[20]

^a^Clinically significant differences ≥ 5 points [29]

^b^*p* value assessed with independent samples *t* test

^c^*p* value assessed with paired samples *t* test

### Analysis of groups based on indication for surgery and histopathology

SF-36 scores were compared in patients with benign functional and benign non-functional tumours before and after adrenalectomy, respectively. Patients with functional tumours had lower HRQoL in physical domains before adrenalectomy than patient with non-functional tumours; physical component summary functional tumours median 33.1 (range 17.1–62.9) vs. non-functional 44.2 (20.0-66.5) *p* = 0.018 (Table 3). Postoperatively, HRQoL was similar in the two groups of patients.

**Table 3:** SF-36 scores in patients with benign functional and benign non-functional adrenal tumours before and at 1 year after adrenalectomy respectively. Median and range are shown

	Preoperative			Postoperative		
	Functional (*n* = 29)	Non-functional (*n* = 15)	*p* value^b^	Functional (*n* = 29)	Non-functional (*n* = 15)	*p* value^b^
Physical functioning	60 (10–100)	85 (10–100)	0.123	80 (10–100)	85 (0–100)	0.236
Role-physical	0 (0–100)	75 (0–100)	0.010^a^	100 (0–100)	100 (0–100)	0.507
Bodily pain	41 (10–100)	84 (0–100)	0.016^a^	84 (21–100)	75 (0–100)	0.602
General health	47 (0–87)	67 (5–97)	0.089	67 (0–100)	76 (20–100)	0.287
Vitality	35 (0–100)	60 (0–95)	0.047^a^	70 (5–100)	55 (0–90)	0.775
Social functioning	62.5 (0–100)	75 (0–100)	0.290	100 (0–100)	87.5 (0–100)	0.565
Role-emotional	33.3 (0–100)	66.7 (0–100)	0.398	100 (0–100)	100 (0–100)	0.867
Mental health	52 (0–100)	76 (0–92)	0.219	84 (20–100)	76 (4–100)	0.718
Physical Component Summary	33.1 (17.1–62.9)	44.2 (20.0–66.5)	0.018^a^	47.6 (19.8–57.3)	49.5 (18.9–68.4)	0.402
Mental Component Summary	33.8 (11.8–62.0)	47.1 (12.3–56.3)	0.629	52.7 (16.4–59.8)	50.0 (15.0–59.6)	0.357

^a^Clinically significant difference ≥ 5 points [29]

^b^*p* value assessed with Mann-Whitney *U* test

Differences between malignant and benign non-functional tumours were investigated. A lower HRQoL was only found for physical functioning in malignant tumours after adrenalectomy; malignant tumour median 67.5 (range 0-95) vs. benign non-functional tumour 85 (0–100), *p* = 0.049 (Supplementary Table 2).

Patients with benign functional tumours reported significantly improved HRQoL in all dimensions after adrenalectomy: Physical Component Summary 33.1 (17.1–62.9) preoperatively vs. 47.6 (19.8–57.3) postoperatively, *p* = 0.005; Mental Component Summary preoperatively 33.8 (11.8–62.0) vs. 52.7 (16.4–59.8) postoperatively, *p* = 0.004 (Table 4). These improvements were not seen in patients with benign non-functional tumours (Table 5).

**Table 4:** SF-36 scores in patients with benign functional tumours before and at 1 year after adrenalectomy respectively. Median and range is shown

	Preoperative (*n* = 29)	Postoperative (*n* = 29)	*p* value^b^
Physical functioning	60 (10–100)	80 (10–100)	0.011^a^
Role-physical	0 (0–100)	100 (0–100)	0.003^a^
Bodily pain	41 (10–100)	84 (21–100)	0.005^a^
General health	47 (0–87)	67 (0–100)	0.036^a^
Vitality	35 (0–100)	70 (5–100)	0.001^a^
Social functioning	62.5 (0–100)	100 (0–100)	0.024^a^
Role-emotional	33.3 (0–100)	100 (0–100)	0.031^a^
Mental health	52 (0–100)	84 (20–100)	0.001^a^
Physical Component Summary	33.1 (17.1–62.9)	47.6 (19.8–57.3)	0.005^a^
Mental Component Summary	33.8 (11.8–62.0)	52.7 (16.4–59.8)	0.004^a^

^a^Clinically significant difference ≥ 5 points [29]

^b^*p* value assessed with Wilcoxon test

**Table 5:** SF-36 scores in patients with benign non-functional tumours before and at 1 year after adrenalectomy respectively. Median and range is shown

	Preoperative (*n* = 15)	Postoperative (*n* = 15)	*p* value^b^
Physical functioning	85 (10–100)	85 (0–100)	0.161
Role-physical	75 (0–100)	100 (0–100)	0.607
Bodily pain	84 (0–100)	75 (0–100)	0.683
General health	67 (5–97)	76 (20–100)	0.157
Vitality	60 (0–95)	55 (0–90)	0.916
Social functioning	75 (0–100)	87.5 (0–100)	0.391
Role-emotional	66.7 (0–100)	100 (0–100)	0.459
Mental health	76 (0–92)	76 (4–100)	0.089
Physical Component Summary	44.2 (20.0–66.5)	49.5 (18.9–68.4)	0.778
Mental Component Summary	47.1 (12.3–56.3)	50.0 (15.0–59.6)	0.109

^a^Clinically significant difference ≥ 5 points [29]

^b^*p* value assessed with Wilcoxon test

In the six patients operated due to malignant tumours, there was no improvement in either Physical or Mental Component Summary score: Physical Component Summary 41.8 (20.5–53.2) preoperatively vs. 34.7 (16.2–53.9) postoperatively, *p* = 0.116; Mental Component Summary 54.5 (32.1–61.7) preoperatively vs. 56.6 (36.5–60.9) postoperatively, *p* = 0.345 (Supplementary Table 2).

When comparing patients with benign functional tumours with the general Swedish population, they had significantly lower SF-36 scores both before and after adrenalectomy (Table 6).

**Table 6:** SF-36 scores in patients with benign functional tumours before and at 1 year after adrenalectomy, compared with the general Swedish population. Mean and standard deviation is shown

	General population* (*n* = 8930)		Benign functional preoperative	*p* value^b^	Benign functional postoperative	*p* value^b^
Physical functioning	87.9 ± 19.6	(*n* = 29)	59.0 ± 29.7	< 0.001^a^	70.0 ± 29.2	< 0.001^a^
Role-Physical	83.2 ± 31.8	(*n* = 29)	33.6 ± 46.4	< 0.001^a^	63.4 ± 44.9	< 0.001^a^
Bodily pain	74.8 ± 26.1	(*n* = 29)	54.2 ± 29.9	< 0.001^a^	68.9 ± 31.7	0.225
General health	75.8 ± 22.2	(*n* = 29)	50.0 ± 27.0	< 0.001^a^	60.4 ± 27.8	< 0.001^a^
Vitality	68.8 ± 22.8	(*n* = 29)	38.1 ± 30.5	< 0.001^a^	57.1 ± 28.8	0.005^a^
Social functioning	88.6 ± 20.3	(*n* = 29)	60.4 ± 32.4	< 0.001^a^	76.7 ± 35.5	0.002^a^
Role-emotional	85.7 ± 29.2	(*n* = 29)	43.7 ± 46.4	< 0.001^a^	66.7 ± 45.4	< 0.001^a^
Mental health	80.9 ± 18.9	(*n* = 29)	56.6 ± 28.7	< 0.001^a^	74.3 ± 23.7	0.061

*Normative values from the general Swedish population published in the Swedish manual and interpretation guide [20]

^a^Clinically significant difference ≥ 5 points [29]

^b^*p* value assessed with independent samples *t* test

Patients with malignant tumours had lower SF-36 scores in four of eight dimensions before surgery compared with the general population; physical functioning malignant mean ± SD 62.5 ± 29.8 vs. general population 87.9 ± 19.6 *p* = 0.002, role-physical 54.2 ± 51.0 vs. 83.2 ± 31.8 *p* = 0.026, general health 56.0 ± 25.8 vs. 75.8 ± 22.2 *p* = 0.029 and role-emotional 55.6 ± 50.2 vs. 85.7 ± 29.2 *p* = 0.012. The difference remained after adrenalectomy except for the domain role-emotional.

Compared with the general Swedish population, patients with benign non-functional tumours had significantly reduced HRQoL before adrenalectomy except for bodily pain, mean ± SD benign non-functional 77.2 ± 29.2 vs. general population 74.8 ± 26.1 *p* = 0.772. Postoperatively, significant differences remained only in the domains social functioning and role-emotional.

## Discussion

In the last decade, the prevalence of adrenal incidentaloma has increased, making the management of adrenal tumours an important aspect of health care. A positive outcome is not only measured by a survival benefit and cure but also by preventing morbidity and by subjective wellbeing of the patients [30]. There are few data on the impact of adrenalectomy on HRQoL in patients with adrenal tumours. Theoretically, HRQoL in patients with small, benign non-functional tumours should not differ from the normal population.

In this cohort of consisting 50 patients with a variety of functional and histological diagnosis, HRQoL improved after adenalectomy. However, compared with the Swedish general population, the patients reported lower SF-36 values both preoperatively and postoperatively. These differences were also deemed clinically significant with more pronounced differences preoperatively.

Patients with benign functional tumours reported lower HRQoL preoperatively when compared with patients with benign non-functional tumours.

Interestingly, no major differences could be detected between patients with malignant and benign non-functional tumours. Thus, patients with benign non-functional tumours reported lower SF-36 scores in mental dimensions and patients with malignant tumours in physical dimensions. The psychological aspects of the disease, e.g. anxiety and fear of malignant disease, may affect HRQoL. Also, small abnormalities in hormonal secretion could be associated with poor health outcomes [31, 32]. In the study cohort, no patients with adrenocortical cancer were detected.

When analyzing the cohort in subgroups, significant improvements in SF-36 scores were only seen in patients with benign functional tumours. The clinical relevance of these results was interpreted as being of moderate or large MID.

Data on HRQoL in patients with tumours in the adrenals are scant [13]. In a case-control study, Kastelan et al. demonstrated reduced quality of life in patients with adrenal incidentalomas compared with age- and sex-matched controls [17]. Brunaud et al. reported lower QoL, estimated by SF-36, in patients with adrenal tumours before adrenalectomy and at 6-weeks follow-up compared with the French general population [16]. During this short time frame, no significant improvement after adrenalectomy could be observed. Previous reports that focused on patients with subclinical Cushing’s syndrome (SCS) reported that adrenalectomy led to an improvement in HRQoL [31, 33]. Similar results have been shown for primary aldosteronism with Conn’s adenoma [18, 34, 35]. These results are in agreement with the results in the previous study. Thus, patients with functional tumours benefited from adrenalectomy with an improvement in HRQoL as estimated by SF-36. However, statistical significance does not equal clinical significance. Clinical significance is rarely commented on in reports on QoL in patients with adrenal tumours [13]. In the present investigation, clinical significance was defined according to Osoba et al. [29]. The results showed that minimal important differences mirrored statistical significance with large differences in SF-36 preoperatively compared with the general Swedish population and with large improvements in HRQoL in patients with benign functional tumours postoperatively.

There are some limitations to the present study. General instruments like SF-36 might not be sensitive enough to grasp the full perspective of patients with a variety of adrenal tumours. Furthermore, SF-36 is a generic instrument, i.e. not disease-specific. Ideally SF-36 should be combined with a tailored questionnaire depending on diagnosis for a greater in-depth knowledge of HRQoL. However, at present, disease-specific instruments are only available for patients with Cushing’s disease [13].

Comorbidity could be associated with poorer HRQoL. These variables were not taken into consideration in the present study. No detailed long-term follow-up was performed after the first outpatient visit 4–6 weeks after adrenalectomy. Only 37% of patients undergoing adrenalectomy during the time period were included in the study. This could lead to inclusion bias. However, this patient group was not different compared with the patients not included the same period.

However, patients with non-functional cortical tumours have been suggested to have an increased risk for the so-called metabolic syndrome, including hypertension and diabetes and cardiovascular risk [8–10, 36, 37], and an improvement of some aspects may be suggested after adrenalectomy [11]. If so, this does not seem to be reflected by an improvement in HRQoL, as shown in the present study.

The strength of the present study is that HRQoL was measured with the well-validated questionnaire SF-36, and results were compared with a sample of the general Swedish population as a reference group. Follow-up was performed at 12 months postoperatively when laboratory and clinical stabilization could be expected.

## Conclusion

Within the context of these limitations, the present study shows that adrenalectomy improved HRQoL in patients with benign functional tumours. Adrenalectomy did not improve HRQoL in patients with benign non-functional tumours or malignant tumours.

## Electronic supplementary material

ESM 1 (DOCX 146 kb)
