# Supplementary material for: Health-related quality of life in patients undergoing adrenalectomy: report from a Swedish National Audit
Source: Langenbecks Arch Surg. 2019 Nov 26;404(7):807–14. doi: 10.1007/s00423-019-01844-4 (PMC6908554; doi:10.1007/s00423-019-01844-4)
Supplement: Supplementary file 1 — (DOCX 146 kb) [file 423_2019_1844_MOESM1_ESM.docx]

Authors:

Lo Hallin Thompson (1)

Erik Nordenström (1)

Martin Almquist (1)

Anders Bergenfelz (1)

Title:

Health Related Quality of Life in patients undergoing Adrenalectomy; report from a Swedish National Audit.

Affiliations and adresses:

1 Department of Surgery, Skåne University Hospital, 22185 Lund

Corresponding author:

E-mail: lo.hallin-thompson@med.lu.se

Tel: +46708218513

Fax: +4646172335

|  | Cohort  (n=50)  Per cent | Non-included  (n=111)  Per cent | p-value |
| --- | --- | --- | --- |
| Age (year, mean ± SD) | 60 ± 12.7 | 60 ± 14.2 | 0.897* |
| Female | 52 | 62 | 0.153 |
| Detection |  |  |  |
| Incidentaloma | 70 | 77 | 0.262 |
| Hormon | 30 | 23 | 0.262 |
| Indication for surgery |  |  |  |
| Hormonal | 54 | 56 | 0.776 |
| Non-hormonal | 46 | 44 | 0.776 |
| Suspected malignancy | 8 | - |  |
| Metastasis | 14 | - |  |
| Size >4cm | 24 | - |  |
| Histopathology |  |  |  |
| Malignant | 12 | 15 | 0.535 |
| Phaeocromocytoma | 24 | 20 | 0.495 |
| Other benign | 4 | 9 | 0.152 |
| Adrenal cortical adenoma | 52 | 45 | 0.322 |
| Adrenal cortical hyperplasia | 4 | 8 | 0.234 |
| Myelolipoma | 4 | 3 | 0.152 |

Supplementary Table 1. Differences between the final cohort and not-included patients undergoing adrenalectomy during the study period.

p-value assessed with Chi-square test

* p-value assessed with independent samples t-test

Supplementary Table 2. SF-36 scores in patients with malignant and benign non-functional tumours before and at one year after adrenalectomy respectively. Median and range is shown.

|  | Preoperative |  |  | Postoperative |  |  |
| --- | --- | --- | --- | --- | --- | --- |
|  | Malignant (n=6) | Benign non-functional (n=15) | p-value^b^ | Malignant (n=6) | Benign non-functional (n=15) | p-  value^b^ |
| Physical functioning | 70 (10-95) | 85 (10-100) | 0.310 | 67.5 (0-95) | 85 (0-100) | 0.049^a^ |
| Role-Physical | 62.5 (0-100) | 75 (0-100) | 0.589 | 62.5 (0-100) | 100 (0-100) | 0.461 |
| Bodily pain | 72 (41-100) | 84 (0-100) | 0.440 | 46 (31-100) | 75 (0-100) | 0.291 |
| General health | 60 (15-87) | 67 (5-97) | 0.532 | 56 (15-77) | 76 (20-100) | 0.147 |
| Vitality | 67.5 (15-100) | 60 (0-95) | 0.389 | 62.5 (5-100) | 55 (0-90) | 0.876 |
| Social functioning | 100 (50-100) | 75 (0-100) | 0.324 | 100 (50-100) | 87.5 (0-100) | 0.353 |
| Role-Emotional | 66.7 (0-100) | 66.7 (0-100) | 0.967 | 100 (0-100) | 100 (0-100) | 0.850 |
| Mental health | 92 (48-100) | 76 (0-92) | 0.118 | 86 (64-100) | 76 (4-100) | 0.240 |
| Physical Component Summary | 41.8 (20.5-53.2) | 44.2 (20.0-66.5) | 0.312 | 34.7 (16.2-53.9) | 49.5 (18.9-68.4) | 0.099 |
| Mental Component Summary | 54.5 (32.1-61.7) | 47.1 (12.3-56.3) | 0.087 | 56.6 (36.5-60.9) | 50.0 (15.0-59.6) | 0.083 |

^a^ Clinically significant difference ≥5 points[30]

^b^ p-value assessed with Mann-Whitney U test


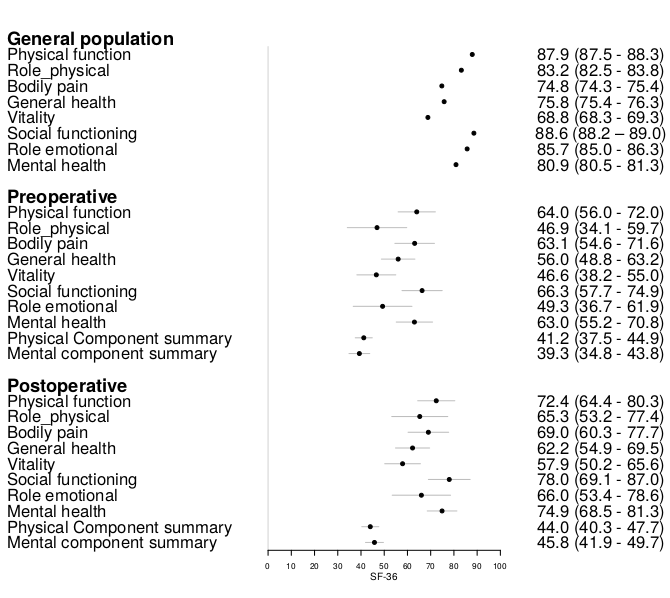


Supplementary Figure 1. Pre- and postoperative SF-36 scores for the cohort in relation to the general population. Mean value (95% confidence interval).
